# The talent project and the validation of standards for the identification of student-athlete talent

**DOI:** 10.3389/fspor.2025.1580625

**Published:** 2025-05-12

**Authors:** Domenico Savio Salvatore Vicari, Antonino Bianco, Martina Albanese, Laura Capranica, Mojca Doupona, Epameinondas Koutavelis, Georgia Alexandrou, Olia Tsivitanidou, Tea Gutović, Ivica Zelić, Dražen Čular, Dino Mujkic, Siniša Kovač, Damira Vranesic- Hadzimehmedovic, Zoran Milanović, Nenad Stojiljković, Nemanja Stanković, Ana Kezić

**Affiliations:** ^1^ Department of Neuroscience, Biomedicine and Movement, University of Verona, Verona, Italy; ^2^ Sport and Exercise Sciences Research Unit, Department of Psychology, Educational Science and Human Movement, University of Palermo, Palermo, Italy; ^3^ Department of Movement, Human and Health Sciences, University of Rome “Foro Italico”, Rome, Italy; ^4^ European Athlete as Student (EAS) Network, Ghaxaq, Malta; ^5^ Department of Sport Sociology and History, Faculty of Sports, University of Ljubljana, Ljubljana, Slovenia; ^6^ Health Promotion and Wellbeing Unit, KMOP Education and Innovation Hub, Athens, Greece; ^7^ School of Sciences, University of Central Lancashire Cyprus, Nicosia, Cyprus; ^8^ Faculty of Humanities and Social Sciences, University of Split, Split, Croatia; ^9^ Center of Excellence of Split Dalmatia County, Split, Croatia; ^10^ Faculty of Kinesiology, University of Split, Split, Croatia; ^11^ Faculty of Sport and Physical Education, University of Sarajevo, Sarajevo, Bosnia Herzegovina; ^12^ Faculty of Sport and Physical Education, University of Niš, Niš, Serbia; ^13^ Science and Research Centre Koper, Institute for Kinesiology Research, Koper, Slovenia; ^14^ Incubator of Kinanthropology Research, Faculty of Sports Studies, Masaryk University, Brno, Czechia

**Keywords:** dual career, talent, coaches, teachers, standards

## Abstract

**Introduction:**

The aim of the TALENT project is to promote equality in education, prevent exclusion, support dual careers (sport and school), create new role models for the benefit of young talents and prepare them for lifelong learning and professional sport from an early age. It is promoted by a European consortium of 7 partner institutions and runs from December 2022 to May 2025. It consists of five work packages. In the first work phase, developing the WP2 (from December 2022 to October 2023), under the coordination of UNIPA, NIS University, KMOP and EAS standards for talent recognition were identified and validated.

**Methods:**

Initially, 12 focus groups were conducted with teachers (77 teachers) and coaches (73 coaches) on creating talent identification standards; subsequently, workshops were held with dual career experts to validate these standards. This was a key piece of work that enabled the establishment of clear guidelines and protocols to identify and support talented young people in their dual careers.

**Results:**

A final list of 41 shared statements was identified: 20 related to teachers and 21 related to coaches. For example, teachers emphasized the need for multidisciplinary approaches and early identification of talent, while coaches underlined the importance of psychological readiness and collaboration with schools and families.

**Discussion:**

These statements not only provide structured reference points for talent identification but also highlight actionable needs across educational and sport systems. As such, they represent a solid foundation for developing standard operating procedures in talent recognition and dual career support.

## Introduction

**Figure 1:**
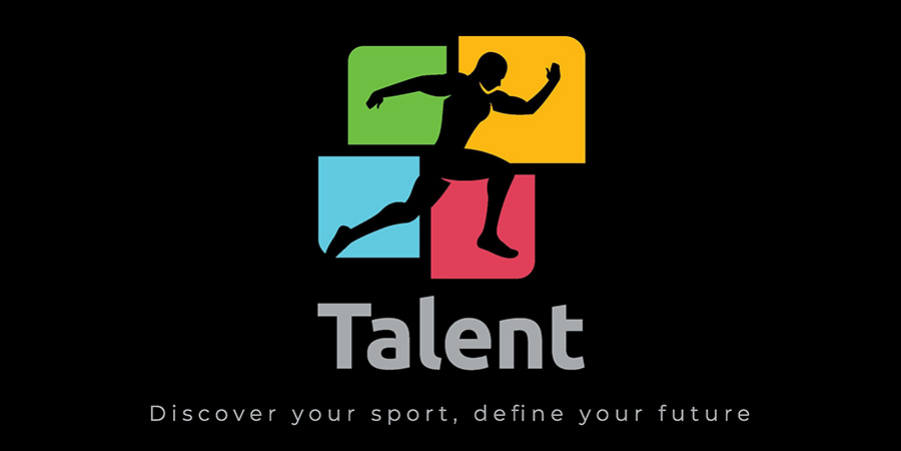
Official logo of the TALENT project.

**Figure 2:**
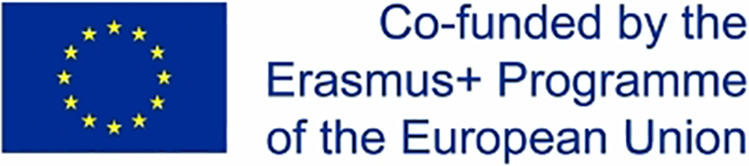
The Erasmus+ logo.

Reconciling high-level sporting activity with education is one of the main problems of the student-athlete who often risks abandoning one of the two activities or not practicing them successfully (1–3). The Erasmus + project Cloud Based Education for Creative Sports Talent^1^ (TALENT) supports dual careers and prevents sports and school dropouts by working on the development of the system for young talents (Figures 1, 2). The partner composition is diverse and thus covers all necessary areas of the TALENT project activities, outputs and results. The consortium consists of seven partner institutions: Higher Education Institutions (University of Palermo and University of Nis); Research center (Center for Social Innovation); Education institution Centar izvrsnosti Splitsko-dalmatinske županije (CI SDZ), and NGOs Kentro Mermnas Oikogeneias Kai Paidiou (KMOP) and Sarajevo Meeting of Culture (SMOC) as well as The European Athlete as Student (EAS) network established with main aim to to support European athletes in combining high performance sport and education. Project aim is to promote equal education, preventing exclusion, supporting Dual Careers, creating new pattern of behavior for the benefit of young talented children's and preparation for Long Life learning and professional sport from childhood. TALENT Project focuses on standards for recognition and awareness increase about sporting talent and the spread of the dual-career concept from the first grades of school talented sport youths from 12 to 16.

To achieve proficiency and to perform at the highest level, talented sports or performing arts youth need to engage in a minimum of 10 years, or 10,000 h of deliberate practice in their chosen field, which requires an early childhood skill development through specific and demanding quality training (4–6). In the developmental years, talented youth encounter several challenges in accommodating concurrent and reciprocal interaction between the demands of school and training commitments (i.e., dual career) (7, 8). Even though children hold the burden of developing their talent, several stakeholders have been identified as supportive entourage at the meso (e.g., parents, peers, teachers/employers, coaches, sport managers), the macro (e.g., sport clubs/federations, educational institutions, and labour market), and the policy (e.g., national and European governing bodies) levels of dual career paths (8–13).

Despite not a single person, institution, or specific context have the main responsibility to help youth improving their personal resources, parents, coaches, and teachers have been recognized a crucial supportive role in enhancing the talented youth's motivation by providing role-models, encouragement, financial support, and logistics (14). However, teachers, coaches, and parents need to be aware and ready to cooperate for fine-tuning their support in relation to the different challenges of dual career paths (15, 16). In fact, it is important to understand specific talent development trajectories. Therefore, to ensure a dual career success the various interested groups involved with nurturing youth need to establish a solid alliance in relation to the specific dimension of talents. The aim of this study was to address the existing gap in dual career research by developing and validating a set of context-sensitive standards and guidelines for talent identification among student-athletes, with the broader objective of strengthening early recognition practices and systemic support across educational and sporting domains.

## Materials and methods

During the first project work phase (WP2) the standards for talent recognition were identified and validated. To ensure diverse perspectives, separate focus groups were held with teachers and coaches during the initial phase of standard development for talent identification, and subsequently, a workshop was organized with dual career experts for the validation of the aforementioned standards. The thematic areas explored during the sessions included participants' professional backgrounds and experiences, current practices and strategies for talent identification in youth sports, and the criteria they consider relevant when assessing children's potential. Further topics addressed included the availability and perceived usefulness of existing guidelines or protocols, the role of collaboration between professionals, the involvement of families in the identification process, and the challenges faced in supporting talented children. Finally, participants were invited to provide suggestions for improving existing systems and to share additional insights based on their practical experience. This stage runs from December 2022 to October 2023, under the coordination of UNIPA, NIS University, KMOP AND EAS standards for talent recognition were identified and validated. Initially, 12 focus groups were conducted with 77 teachers and 73 coaches on creating talent identification standards; subsequently, workshops were held with dual career experts to validate these standards. In each country, the teachers and coaches participated in the focus group using their native language and the final results were translated into English.

A final list of 41 shared statements was identified: 20 statements related to teachers and 21 statements related to coaches on talent identification. This list of statements could help coaches or teachers identify talent. The study was conducted in accordance with the recommendations of the Declaration of Helsinki for the involvement of people in research and was approved by the Ethics Committee of the Faculty of Sports and Physical Education of the University of Nis (04-215/2).

### Focus groups

#### Participants

Each partner, except 1 (EAS^2^), conducted two focus groups: one with at least 12 sports coaches and one with at least 12 teachers. To be recruited, the coaches had to have a background of at least 5 years of field activity. We have tried to recruit a fair number of team and individual sports coaches. As regards teachers, to be recruited, they also had to have at least 5 years of school activity at different levels of education. One hundred fifty (150) participants contributed their valuable insights and experiences, with 77 teachers/educators and 73 coaches. The findings from the focus group discussions in Greece, Italy, Serbia, Bosnia and Herzegovina, Cyprus, and Croatia collectively shed light on the complex landscape of identifying and supporting talented student-athletes. While each country provided unique insights based on its specific context, certain common themes and needs emerged across these diverse settings.

#### Procedures

The focus group interview consisted of 8 open-ended stimulus questions. Questions for the focus group with teachers are shown in Table 1. Questions for the focus group with coaches are shown in Table 2. The duration of the focus groups was 60 min. The focus group was conducted in a comfortable room in a convenient location, responsive to the needs of the participants. Participants were seated in a circle with some participants connected remotely as agreed at the TPM in Palermo by all partners. Before proceeding, all participants signed an attendance and consent form. All participants were notified that the meetings were being recorded. Each focus group was led by a moderator and an assistant moderator. The moderator promoted the discussion and debate, and the assistant took notes. All partner countries used the same questionnaire model for each target group (coaches and teachers) in order to have a common basis for the analysis of the results. Once the focus groups were carried out, a report was created for each partner summarizing the results collected from the focus groups. By collecting this data, it was possible to comprehensively understand the results of the focus groups and use them to identify standards for gifted children.

**Table 1:** Questions for teachers.

Questions for teachers	Research topic
1. Can you briefly introduce yourself, how long have you been working with children, and what is your experience as a teacher?	Introduction and background
2. How do you evaluate the current methods of detecting sports talents in the school environment? 3. What criteria or characteristics do you consider when identifying talented children (in sports) in your field? Are there any specific indicators or behaviours that catch your attention? 4. Are available any guidelines or protocols to you for detecting and supporting sports talents in school? What additional resources or training would you like to have?	Identifying talent
5. What strategies do you use to identify children's sports talents? Do you involve parents or guardians in the process? Any best practices?	Observation and assessment
6. Do you communicate with other coaches/teachers to identify and support talented children? If yes, are there any challenges you face in communicating effectively?	Collaboration and communication
7. What obstacles do you face when identifying or supporting talented children? Do you have any techniques to overcome them?	Overcoming obstacles
8. Are there any suggestions you would like to make for establishing and improving the system or any additional insights you would like to share?	Closing thoughts

**Table 2:** Questions for coaches.

Questions for coaches	Research topic
1. Can you briefly introduce yourself, how long have you been working with children, and what is your experience as a coach?	Introduction and background
2. How do you evaluate the current methods of detecting sports talents in your sport? 3. What criteria or characteristics do you consider when identifying talented children in your field? Are there any specific indicators or behaviours that catch your attention? 4. Are available any guidelines or protocols to you for detecting and supporting sports talents? What additional resources or training would you like to have?	Identifying talent
5. What strategies do you use to identify children's sports talents? Do you involve parents or guardians in the process? Any best practices?	Observation and assessment
6. Do you communicate with other coaches physical education and health teachers to identify and support talented children? If yes, are there any challenges you face in communicating effectively?	Collaboration and communication
7. What obstacles do you face when identifying or supporting talented children? Do you have any techniques to overcome them?	Overcoming obstacles
8. Are there any suggestions you would like to make for establishing and improving the system or any additional insights you would like to share?	Closing thoughts

### Workshop

To further emphasize these statements’ significance and reach a consensus on their relevance, importance, and necessity, a pivotal workshop was convened in Gaeta (Latina) on 3 and 4 October 2023, by the project partner European Athlete Student (EAS). The EAS Conference represents the annual meeting point for international experts to discuss and share policies and initiatives to support the compatibility of sports and academic/work careers (e.g., dual career) of the sportspersons, including lectures, workshops, and round tables on relevant aspects of dual career.

#### Participants

A group of 25 people (academics, members of International Sports Organizations, members of the Paralympic Committee, and members of the EAS) international experts on the topic of dual careers participated in this workshop.

#### Procedures

The workshop was held on 3 October 2023 from 17:00 to 18:00. The workshop was introduced by two contributions framing the talent project and the pathway developed within WP2. This introductory phase was followed by the questioning of the experts. Through the Mentimeter software and the sharing of two QR codes with the audience, the experts had the opportunity to evaluate, according to the RELEVANCE criterion, each statement produced. First, the statements taken from the focus groups conducted with the teachers were evaluated, and then the statements taken from the focus groups conducted with the coaches.

Before going on to present the results of the workshop, we would like to briefly illustrate the process that led to the formulation of the statements to be submitted to the experts for validation. The partners discussed the statements through a shared excel file in which each partner produced its own statements concerning the results of the national focus groups developed.

They then followed the standards developed by each partner.

The statements above were synthesized and analyzed. The criteria followed in constructing the summary statements were recurring statements between the partners, complementary aspects on talent identification, summary, affirmative form.

A final list of 41 agreed statements were identified: 20 statements related to teachers and 21 statements related to coaches on talent identification. Analysis of the results led to a reorganization of the standards in order of relevance. No standard was excluded as no statement was deemed “not relevant”.

## Results

### Focus group with coaches

The focus groups in Greece, Italy, Serbia, Croatia, Cyprus, and Bosnia and Herzegovina provided valuable insights into the challenges and strategies for identifying and nurturing talented children in sports. Common themes emerged, underscoring the importance of a holistic approach to talent development. Across these countries, coaches faced challenges related to the lack of formal guidelines and protocols for talent identification, parental expectations, and coordination with the school system. They recognised the significance of character traits, early exposure to sports, and psychological readiness in identifying talent. Coaches also emphasised the need for better infrastructure, cooperation among sports clubs, and multi-stakeholder collaboration involving parents, schools, and sporting organisations. To improve the talent identification process and support talented children effectively, coaches called for standardised parameters and standards, dual career options, and measures to ensure the long-term development of young athletes. While each country had unique experiences and challenges, the collective insights highlighted the importance of addressing these issues to create an environment where young athletes can thrive and fulfil their potential in sports and education.

Each partner developed standards, in the form of statements, summarising the content of the focus groups conducted. An overview of the results country by country is shown in Table 3.

**Table 3:** Standards developed by each partner.

Standards developed by each partner		
Teachers statements SMOC	1	Proper guidance enhances the effectiveness of athletes who can strategically utilize their potential in physical activities
	2	Important to support, select and properly direct children towards the more suitable sport
	3	Evident lack of a talent recognition system, which poses a significant problem in identifying and supporting gifted children
	4	Establish a multidisciplinary approach to talent identification
	5	Necessary collaboration between sports experts, educational personnel, and psychologists
	6	Better understanding of children's needs and facilitates among teachers needed to support talented children
	7	Should special curricula be designed for talented children
	8	An insufficient number of grades is accumulated during a semester due to participation in sport contests (who is to blame? – the children, teachers, parents, school, coaches?)
	9	The information flow requires better communication between teachers, pupils/students and parents
	10	Should the government think about special schools for talented children
	11	It all comes down to personal success of individual children - no talent recognition mechanisms exist
	12	Evident lack of knowledge of teachers on the topic of talent recognition (organise trainings, seminars, etc)
Coaches statements SMOC	1	Expressed frustration about the lack of support and understanding from teachers, who often failed to recognize the demands and commitments faced by young athletes
	2	Conflicts between school obligations and sports training or competitions, resulting in talented athletes feeling discouraged and a high dropout rate
	3	Need for Improved communication and cooperation
	4	Coaches proposed better communication channels and cooperation between schools, sports clubs, coaches, and parents
	5	Creating a mutual understanding and support system that allows young talents to pursue their athletic ambitions
	6	The coaches stressed the need for training and awareness programs for teachers to enhance their understanding of the specific needs and challenges faced by talented student-athletes
	7	A more supportive environment should be established for young talented athletes
	8	Synchronization with the school system is necessary (not that coaches and clubs can always influence/decide on competition dates)
	9	Absence of clear rules, protocols, and instructions for identifying and developing talented children in sports
	10	Establishment of specific parameters and standards to guide talent identification at different age groups
	11	Challenges related to infrastructure and financing, as the existing system often prioritized short-term results over the long-term development of talented children
	12	Better infrastructure to provide optimal conditions and a revised financing system that recognizes and supports talent development
	13	Improving coordination, communication, and understanding among teachers, coaches, parents, and sports clubs
	14	Improvements in school-sport club communication and cooperation, including dual career options
	15	Expectations and ambitions of some parents often overshadow the needs and desires of the children themselves
	16	Parents should also be involved in the process of promoting and supporting talents
Teachers statements UNIPA	1	Difficulty in defining one's role in talent recognition
	2	Extracurricular engagement
	3	Awareness of unwillingness to deal with the topic
	4	Pay attention to the spontaneous actions of students
	5	Every student has a talent
	6	Synergy between teacher, family, community
	7	Inadequate school structures
	8	Need for sports technicians or qualified persons in the teaching team
	9	Need for economic incentives
	10	Multidisciplinary experimentation
	11	Systemic change in mentality
	12	Making investments in infrastructure and school buildings
Coaches statements UNIPA	1	There is no specific and systematic approach for the identification of sporting or other talents in the sporting environment
	2	Each sport travels different paths although they are considered complementary
	3	Each coach identifies and manages subjectively students' talent, using their instinct and personal perceptions
	4	Each coach identifies and manages students' talent with professionalism and competence
	5	Time for talent to flourish is necessary
	6	Sports technicians' experience is crucial
	7	Lack of resources
	8	Few trainers
	9	The activity must be differentiated, and the talent must be carefully monitored and cultivated; on the other hand, space and attention must be given to everyone equally.
	10	Facilities are often lacking
	11	Creating a network outside the locality
	12	Talent is “a gift from mother nature”
	13	A talent is such to the extent that there is a good coach who knows how to identify, manage, and accompany it.
	14	Talent is proportionate to the commitment and perseverance a talent puts into his or her training project.
	15	Training is a special pedagogical and sporting process.'
	16	Commitment and genetics are crucial, but they are not enough
	17	Talent identification occurs differently depending on the type of sport
	18	Talent is someone who can “replicate the technical gesture in a natural way”
	19	Talent also depends on character and mental ability
	20	Stubbornness, courage, imagination, grit and optimism, perseverance, humility, and the desire to grow are all transversal elements that play a part in the formation of talent
	21	Acquiring self-awareness, both of weaknesses and strengths, is crucial
	22	There are those who have a natural inclination and those who need more training, but it is all in the management of talent
	23	Becomes imperative to coach by skills not by age
	24	The family is often a critical point even if it is considered important
	25	A team must be created to support the trainee athlete.
	26	Long-term planning is successful
Teachers statements KMOP	1	We cannot discuss sports facilities in public schools when there are still classrooms in containers or building basements
	2	We are not trained to recognise talented students
	3	Each professional should stick to their expertise and talented students should be monitored by their coaches
	4	Collaboration with the school and the sports clubs is rare
	5	There is no funding to collaborate or organise mutual activities
	6	It's difficult to recognise talent
	7	We don't have tools/resources to recognise talented students
	8	Need of seminars on the active role of schools/educators in students' dual careers
	9	Seminars towards educators about the benefits of sports in student's life
	10	Collaboration between schools-sports clubs is important
	11	Need of professionals to monitor and support students and their skills through an assessment
	12	Need for strengthening public sports schools' operation
	13	Implementation of specific programs in schools dedicated to talented athletes and their support
Coaches statements KMOP	1	There is no long-term program to support young athletes throughout their developmental and athletic stages and their inclination to be detected and supported
	2	There are no official programs and methodologies in recognising talent
	3	There are initiatives that allow coaches to observe the minor participants in different sports and inform their parents about their kid's inclinations
	4	Coaches need to use their personal experience to monitor and recognise talent
	5	Most sports are considered a hobby for most parents, so they do not care about standardised methodologies and strategies
	6	In case coaches detect a talent, they talk to parents, but some sports are undervalued and they do not pursue it
	7	it is difficult for such methodologies to be applied, due to the lack of the necessary equipment, time and staff
	8	I rarely speak with educators about sports and talent
	9	I usually communicate with the parent and the parent with the educator and vice versa
	10	Adaptation of methodologies/best practices from abroad to national context
	11	Training of trainers on soft skills deriving from sports psychology
	12	Primary measurements in schools from sports educators in basic sports skills (e.g., speed, balance, and strength)
	13	Regular follow-up tests and measurements of all athletes
	14	Development of a common tracking system per sport
	15	More accurate and faster methodologies
	16	Introducing a broader range of sports in the formal educational system to provide children with the opportunity to explore and familiarize themselves with various sports
	17	Close collaboration between parents-school-coaches for the co-creation of a realistic plan for talented athletes
	18	Specific training programs in talent identification for physical education teachers
	19	Human and financial resources to support relevant protocols and procedures in the sports club
Teachers statements CI SDZ	1	There is no specific and systematic approach for detecting sports or any kind of other talent in school environment.
	2	Puberty as a key dropout age from any activity, necessity to work more to avoid it
	3	General lack of funding of any and structural restrictions to any additional activities
	4	Teachers rely on their expert eye to detect talent
	5	Talented children always ask clever questions
	6	Talented children have logical reasoning that is over developed for their age.
	7	More active children are always more talented for sports in general
	8	Talent identification and development as a marathon process, not a sprint.
	9	Talented children are fast learners, but they need continuation in their work and only with constant work and support their talent will develop
	10	Public awareness on these topics is very low
	11	Communication with children is key
	12	Communication with parents is necessary in order to be on same page for child's development.
	13	Educators need to take into an account parents' perspective and work alongside them.
	14	Collaboration between school and sport clubs depends mostly on good will of the individuals within organisations.
	15	Talented children need to be taught they need to work on their talent
Coaches statements CI SDZ	1	Coaches are not familiar with any specific methods or any system for detecting sport talent.
	2	Coordination ability is an ability that makes a difference in any sport.
	3	System of discovery of talent is for most of them often based on putting children in more encouraging environments
	4	Biological characteristics are not enough to detect talented children
	5	Character is key factor as well as children's motivation
	6	Children who are (based on some preliminary talent identification) proclaimed as “stars” soon lose interest and motivation
	7	Necessity for better and more equipped infrastructure in order to have more better conditions to be able to direct more attention towards talented individuals
	8	Need for better cooperation within their specific sports with other clubs as well as with other experts
	9	Talent identification is a long-term process and that it depends on coaches' understanding children's needs.
	10	Development of children has to be simultaneous process with the development of parents as their support system
	11	Biggest issue (apart from infrastructure) is bad synergy with school system
	12	Would like to have specific parameters and standards for identification of sport talents in specific age.
Teachers statements CSI	1	Talent recognition is mainly based on daily observations of students' performance in the class
	2	Talent recognition is also based on performance testing (e.g., tests, competitions)
	3	Athletic, or sports talents often surface during informal assessments and competitions conducted within the school setting.
	4	Certain behavioural traits appear in children with a specific talent (in any field) such as confidence, enthusiasm, patience, and commitment to the skill or subject in which they excel.
	5	Children who show an exceptional interest in a specific subject often require less effort to meet established benchmarks
	6	It is important to offer opportunities to children to exhibit their talents.
	7	A talent show organized at the schools was a good incident for teachers to uncover talents they had previously been unaware of.
	8	Talent identification sometimes occurs unexpectedly; for instance during a conversation with parents
	9	Teachers often go beyond conventional curricular requirements to offer additional support to gifted students
	10	Extracurricular activities offered through afternoon clubs provide an alternative yet equally significant platform for talent cultivation
	11	For nurturing talent, especially in sports, the collaborative efforts between teachers and coaches play a pivotal role
	12	There are however ongoing challenges in executing a seamless collaborative approach between teachers and coaches; better communication is required
	13	Communication between teachers and parents or guardians emerges as a crucial element in supporting talents
	14	In the secondary educational level of the school, the operation of a “sport school” program offers a tailored schedule to talented athletes
	15	It is a challenge to balance individualized student needs with the advancement of gifted/talented learners in the classroom setting
	16	Another challenged is the limited parental involvement, as teachers find it challenging to gain parental support for nurturing their children's talents
	17	There is a need for specialized approaches and interventions specifically designed for nurturing talent
	18	There is a need for strong communication with external experts or coaches for specialized talent development
Coaches statements CSI	1	A key approach for talent identification involves using coaches' observations of children's performance
	2	Another indicator is how quickly a child can learn a new skill or technique. Rapid acquisition of skills can be a sign of natural aptitude
	3	The use of specialized tests to measure quantifiable traits in athletes, varying by sport, for talent identification
	4	Examples include the time taken to execute a particular exercise or drill and the success rates achieved
	5	Another example from football, if a child consistently scores average or above in these tests, it might indicate that they have a significant talent
	6	In swimming, a first sign of talent is a child's lack of fear of water, followed by how well they follow the instructor's guidelines
	7	Various tests are used that measure agility, explosiveness, speed, and other ergometric measurements for talent identification
	8	Personality traits of a child or athlete were also identified as crucial indicators of potential, e.g., leadership skills
	9	Talent identification in younger children should emphasize on basic motor skills and cognitive traits as initial indicators, e.g., proper walking, correct body posture, appropriate running techniques, reaction times, changes in direction, hand-eye coordination, and the ability to quickly process and apply new information
	10	There are established norms for every sport, catering both to technical and psychological training
	11	Talent identification is multifaceted, which is not restricted just to physical prowess but also encompasses mental and psychological aspects
	12	Collaboration among coaches is an essential strategy
	13	Still, there is room for improvement in the approaches to identify and support talents in athletics
	14	It is important for the coaches to continuously be updated with information on the subject (talent identification)
	15	The experience of a coach plays a significant role in identifying talent
	16	The existence of a universally applicable tool that could be utilized by any coach without the need for specialized equipment remains a gap
	17	Identifying talent does not ensure its growth. The child's social environment – encompassing family, friends, and teammates – plays a significant part
	18	Also, factors like the nature of the child's daily obligations, the school commitments, and the type of school they attend (i.e., whether it is a sports-school or not), also play a pivotal role
	19	For team sports, the promotion of talent appears to be more challenging when compared to individual sports
	20	Communication with parents and guardians is challenging
	21	The mindset of parents often influences their child's development in a sport
	22	In team sports like football, one challenge highlighted by coaches is to find the correct position for a talented child on the field
	23	Another constraining factor is the quality of the facilities
	24	Parents usually prioritize their child's academic pursuits
	25	For future implementation strategies, coaches showed interest in the development of a universally applicable talent identification tool
	26	There is also a consensus on the need to establish programs that balance both athletic and academic pursuits
Teachers statements FSFV	1	The motivation of students to participate in sports is a major barrier
	2	Technology, financial constraints, and declining discipline were identified as hurdles to sports participation
	3	Cooperation between schools and sports associations boost student involvement
	4	The role of parents as both positive contributors and potential sources of pressure is recognized
	5	The absence of a structured system for identifying sports talents in schools was a key concern
	6	The pilot project involving physical education classes and sports examinations is promising for talent identification and development
	7	Reintegrating sports sections into schools is necessary to instil a passion for sports from a young age
	8	Partnerships between schools, sports associations, and city-level competitions is important for talent development
	9	Teachers supported initiatives like physical education classes in schools with enriched schedules to identify talent
	10	Mass competitions and cooperation between schools and sports associations are welcome to increase student engagement in sports
Coaches statements FSFV	1	The complexity of measuring talent, particularly in complex sports like football, is a major barrier to effective talent identification
	2	The absence of a definitive formula for talent selection is recognized
	3	Challenges related to international standards, attributes like speed and courage, and early exposure to sports are the most frequent
	4	Communication challenges between coaches and teachers and varying perspectives on parental involvement exist
	5	There is a need for improvement, including measurement systems and parental education to optimize talent identification and support young athletes
	6	Performance evaluations during training and competitions, motor skills assessments, and consideration of anthropometric data are key parameters for talent identification
	7	Some teachers were willing to support children in sports competitions, and primary school teachers played a vital role in preparing students with well-developed motor skills
	8	Suggestions for improvement included structured parents' meetings in sports clubs to educate parents and build trust between parents and coaches
	9	Effective strategies included early exposure to sports opportunities in school settings and structured meetings between coaches and teachers
	10	The importance of parental education to encourage a balanced approach and prevent undue pressure on young athletes was recognized

Common findings include the urgent need for teacher training and the call for better school-sport collaboration. These recurring themes reinforce the need for systemic interventions and serve as reference points for policy implementation.

### Focus group with teachers

Across Greece, Italy, Serbia, Cyprus, Croatia, and Bosnia and Herzegovina, the focus group discussions underscored common challenges and unique aspects of identifying and nurturing talented children within their respective education systems. Common challenges included the lack of a structured system for talent recognition, financial constraints, the role of technology in distracting students from physical activities, and the need for improved communication between schools, parents, and sports clubs. Educators in these countries emphasised the importance of early identification of talent and the need for multidisciplinary approaches to support talented children in their dual careers. While each country had its unique context and practices, some shared recommendations emerged. These included advocating for professionalization in coaching, creating opportunities for extracurricular activities, involving parents constructively, and developing systematic approaches to talent identification and development. In conclusion, fostering the development of talented children in sports and other domains requires concerted efforts across the education system, sports organisations, parents, and the wider community. Implementing structured systems for talent recognition and support, along with improving communication and collaboration among stakeholders, are essential steps toward nurturing these young talents' potential and helping them succeed in their chosen fields. The full results are reported in the Table 3.

#### Common themes

• Communication and Cooperation: In all countries, there was a consensus on the necessity of improved communication and cooperation between educational institutions, sports clubs, coaches, and parents to provide holistic support for talented student-athletes.
• Structural Challenges: Participants highlighted challenges related to infrastructure, financing, and resources in both sports clubs and educational institutions. Addressing these structural deficiencies was considered crucial.
• Parental Involvement: The role of parents in the talent development process was acknowledged as crucial but also challenging. Striking a balance between parental support and undue pressure on children was emphasised.
• Standardisation and Training: The need for standardised methods and training programs to streamline talent identification and support processes was a recurring theme.
• The statements above were synthesized and analyzed. The criteria followed in constructing the summary statements were:
• recurring statements between partners,
• complementary aspects on talent identification,
• summary,
• affirmative form.

#### Workshop

Unified statements are according to the explicit criteria and ordered according to the highest score obtained in relation to the “RELEVANCE” criteria and are reported in the Table 4.

**Table 4:** Unified statements achieved in terms of percentage.

Unified statements teacher	Score (%)	Unified statements coaches	Score (%)
1. Investments in infrastructure and school buildings	9	1. Long-term planning is successful	6
2. Specific training on talent recognition by technicians	7	2. Official programs and methodologies in recognising talent	6
3. Multidisciplinary integrated intervention	6	3. Coaches' experience is crucial	8
4. Synergy between teacher and family	6	4. Training is a pedagogical, psychological and sporting process	6
5. Need for tools and resources	6	5. The basic soft skills demanded by educational environments are fundamental	4
6. Raising awareness of dual careers and the benefits of sport	5	6. Regular follow-up tests and measurements of all athletes	6
7. Systematic intervention on the support of the student athlete	5	7. Developing a sport-specific monitoring system	5
8. Economic incentives	4	8. Human and financial resources to support relevant protocols and procedures	8
9. Student observation in the classroom is crucial	4	9. Synergy between coaches and family	7
10. Talent identification is based on testing	4	10. Synergy between coaches and school environments	9
11. Preventive measures school and/or sports drop-out	6	11. Adequate resources and facilities	5
12. Creating opportunities to identify talent	7	12. Genetic component	4
13. Creating a talent show is a good practice	4	13. Mental and temperamental component	4
14. Extracurricular engagement	5	14. Child development is a simultaneous process with parental development as a support system	4
15. Focus on interpersonal communication	4	15. Age-specific parameters and standards	3
16. Balancing individual student needs	5	16. Talent observation is crucial	6
17. Initial and process evaluation are crucial	3	17. The rapid acquisition of skills is a sign of natural aptitude	3
18. Need for specific dual career programmes for schools	5	18. Talent identification is based on standardised tests	2
19. Synergy between teacher and community	3	19. Synergy between coaches from different sports	2
20. Define specific curricula for sports talent	4	20. Basic motor skills and cognitive traits as initial indicators	3
		21. Balance between sporting and educational objectives	5

## Discussion

Standards for the recognition of students’ talent emerged from the course developed. Among other things, the discussion with experts also brought out standards that are more relevant than others. A limitation of the investigation carried out might be that the results arrived at would differ depending on the point of view with which one looks at the issue. This means that probably if the standards had been analysed by the coaches or teachers, different conclusions would have been reached. However, the involvement of the experts, academics and scholars on dual careers could represent a desire to look at the issue through the eyes of those who are *super partes* in order to give relevance to the person experiencing the dual career condition.

From the results of the focus groups (Table 3) and the standards generated (Table 4) it is possible to see some divergence or agreement between the different groups involved in the production of the document. Indeed, these results underscore the importance of recognizing the multifaceted nature of talent identification and development.

The cross-national results, particularly the high relevance scores attributed to statements such as “Creating opportunities to identify talent” (teachers, 7%) and “Coaches” experience is crucial' (coaches, 8%), highlight key priorities for real-world application. These findings provide not only conceptual insights but also practical guidelines that institutions can adapt to enhance dual-career support for young athletes.

Bridging the gap between sports clubs and educational institutions, establishing formal guidelines, fostering cooperation, and providing adequate resources are key steps toward effectively meeting the specific needs of talented children, ensuring their holistic development, and nurturing their academic and athletic potential. The gap between sports clubs and educational institutions was frequently mentioned, with participants from both groups citing communication breakdowns and lack of cooperation These gaps often result in fragmented support systems for talented children and contribute to early dropout risks (17, 18).

The insights gained from these diverse perspectives offer valuable input for future research and policy formulation in the field of talent identification and support.

An additional point worth considering is the potential dynamic nature of standards for recognizing talent, which may evolve over time and differ significantly across cultural and institutional contexts. While the study highlights the value of expert perspectives in defining these standards, it also implicitly underscores the need for inclusive and continuous dialogue involving all relevant stakeholders — including students themselves, families, coaches, and educators. This broader involvement could enrich the framework by incorporating practical insights from those directly engaged in the day-to-day realities of dual careers (13).

Moreover, the tension between objective criteria and the subjective nature of talent identification raises an important methodological challenge. Standardization, while beneficial for comparability and policy alignment, must be balanced with the flexibility to adapt to individual pathways and the diverse trajectories that talented students may follow. In this sense, the dual career model should be seen not only as a structured support system but also as a living ecosystem that needs to be responsive to personal growth, emotional well-being, and long-term aspirations beyond sport or academia alone.

Therefore, the current findings should be seen as a foundation rather than a conclusive endpoint. They serve as a springboard for further interdisciplinary research and dialogue.

## Conclusion

Talent identification plays a key role in sports and school dropout prevention measures. Therefore, validation of these standards provides an opportunity to work in accordance with the SOP (standard operating procedures) methodology, i.e., to think in terms of clear and unambiguous models and information about the meaning of “talent”. Although the concept of “talent” is inherently complex and sometimes ambiguous, the inclusion of concrete examples, such as students showing rapid acquisition of sport-specific skills or consistent demonstration of psychological traits like perseverance, leadership, and adaptability, helps to clarify its meaning.

The project's main takeaway is the urgent need for structured, collaborative, and context-sensitive approaches that address both the sporting and educational needs of student-athletes grounded in the voices of coaches, teachers, and experts across Europe.

The need to create standards for the recognition of talented children on the one hand, it might be limiting to discuss in terms of standards if one assumes the complexity of the issue, the contexts and the person as such (15, 19, 20). However, it turned out to be a necessity to proceed according to the SOP (standard operating procedures) methodology, i.e., to think in terms of clear and unambiguous models and information. Although the phenomenon is hardly verifiable and there is a certain degree of uncertainty, through reasoning mediated by the SOP methodology, secondary bias is excluded. Thus, having concluded that there is no consensus on what is to be understood by the term “talent”, a shared methodology could significantly contribute to the study of, on and for talent (21–23).

## Data Availability

The original contributions presented in the study are included in the article/Supplementary Material, further inquiries can be directed to the corresponding author.

## Footnotes

^1^Funded by the European Union. Views and opinions expressed are however those of the author(s) only and do not necessarily reflect those of the European Union or the European Education and Culture Executive Agency (EACEA). Neither the European Union nor EACEA can be held responsible for them.

^2^The EAS Network is committed to optimising training conditions for athletes/students, but also to securing their future by making education or vocational training possible, so that they have a secure platform from which to start again once their sports career is over. EAS's reach is Europe-wide and has no specific country of reference this is why EAS did not conduct FGs.

## References

[1] Gomez J Bradley J Conway P. The challenges of a high-performance student athlete. Irish Educ Stud. (2018) 37(3):329–49. 10.1080/03323315.2018.1484299

[2] Izzicupo P Di Baldassarre A Ghinassi B Abelkalns I Bisenieks U Sanchez-Pato A Exploring dual career quality implementation at European higher education institutions: insights from university experts. PLoS One. (2022) 17(11):e0277485. 10.1371/journal.pone.0277485 36449451 PMC9710783

[3] Vidal-Vilaplana A Valantine I Staskeviciute-Butiene I González-Serrano MH Capranica L Calabuig F. Combining sport and academic career: exploring the current state of student-athletes’ dual career research field. J Hosp Leis Sport Tour Educ. (2022) 31:100399. 10.1016/j.jhlste.2022.100399

[4] Ericsson KA Krampe RT Tesch-Römer C. The role of deliberate practice in the acquisition of expert performance. Psychol Rev. (1993) 100(3):363–406. 10.1037/0033-295X.100.3.363

[5] Côté J Baker J Abernethy B. Practice and play in the development of sport expertise. In: Tenenbaum G Eklund RC, editors. Handbook of Sport Psychology. 3rd ed. Hoboken, NJ, US: John Wiley & Sons, Inc. (2007). p. 184–202.

[6] Abbott A Button C Pepping GJ Collins D. Unnatural selection: talent identification and development in sport. Nonlinear Dyn Psychol Life Sci. (2005) 9(1):61–88. 15629068

[7] Pummell E Harwood C Lavallee D. Jumping to the next level: a qualitative examination of within-career transition in adolescent event riders. Psychol Sport Exerc. (2008) 9:427–47. 10.1016/j.psychsport.2007.07.004

[8] Stambulova NB Ryba TV Henriksen K. Career development and transitions of athletes: the international society of sport psychology position stand revisited. Int J Sport Exerc Psychol. (2021) 19(4):524–50. 10.1080/1612197X.2020.1737836

[9] Commission E, Directorate-General for Education Y, Sport, Culture. White Paper on Sport. Luxembourg: Publications Office of the European Union (2007).

[10] Commission E, Directorate-General for Education Y, Sport, Culture. EU guidelines on Dual Careers of Athletes—recommended Policy Actions in Support of Dual Careers in High-performance sport—approved by the EU Expert Group “Education & Training in Sport” at its Meeting in Poznań on 28 September 2012. Luxembourg: Publications Office (2013).

[11] Commission E, Directorate-General for Education Y, Sport, Culture. Study on the minimum Quality Requirements for Dual Career Services—Final Report. Luxembourg: Publications Office (2016).

[12] Parliament E, Union D-GfIPot, Guidotti F Capranica L. Qualifications/dual Careers in Sports—research for CULT Committee. Luxembourg: European Parliament (2016).

[13] Stambulova NB Wylleman P. Psychology of athletes’ dual careers: a state-of-the-art critical review of the European discourse. Psychol Sport Exerc. (2019) 42:74–88. 10.1016/j.psychsport.2018.11.013

[14] Gould D Carson S. Life skills development through sport: current status and future directions. Int Rev Sport Exerc Psychol. (2008) 1:58–78. 10.1080/17509840701834573

[15] Capranica L Doupona M Abelkalns I Bisenieks U Sanchez-Pato A Canovas-Alvarez FJ Understanding dual career views of European university athletes: the more than gold project focus groups. PLoS One. (2022) 17(2):e0264175. 10.1371/journal.pone.0264175 35213599 PMC8880749

[16] Capranica L Guidotti F Gonçalves C Blondel L Bovis M Costa R Development of an online multilingual educational programme for parents of dual-career athletes: a participatory design. Front Psychol. (2022) 13:855531. 10.3389/fpsyg.2022.855531 35936254 PMC9354488

[17] Knight CJ Harwood CG Sellars PA. Supporting adolescent athletes’ dual careers: the role of an athlete’s social support network. Psychol Sport Exerc. (2018) 38:137–7. 10.1016/j.psychsport.2018.06.007

[18] Ryba T Stambulova N Ronkainen N Bundgaard J Selänne H. Dual career pathways of transnational athletes. Psychol Sport Exerc. (2015) 21:125–34. 10.1016/j.psychsport.2014.06.002

[19] Gjaka M Tessitore A Blondel L Bozzano E Burlot F Debois N Understanding the educational needs of parenting athletes involved in sport and education: the parents’ view. PLoS One. (2021) 16(1):e0243354. 10.1371/journal.pone.0243354 33471807 PMC7817049

[20] Stambulova N Wylleman P Torregrossa M Erpic SC Vitali F de Brandt K FEPSAC position statement: athletes’ dual careers in the European context. Psychol Sport Exerc. (2024) 71:102572. 10.1016/j.psychsport.2023.102572 38030052

[21] Nikander J Tolvanen A Aunola K Ryba TV. The role of individual and parental expectations in student-athletes’ career adaptability profiles. Psychol Sport Exerc. (2022) 59:102127. 10.1016/j.psychsport.2021.102127

[22] Sargent Megicks B Till K Rongen F Cowburn I Gledhill A Mitchell T Examining European talent development environments: athlete, parent and coach perceptions. J Sports Sci. (2022) 40(22):2533–43. 10.1080/02640414.2023.2172800 36724148

[23] Thompson F Rongen F Cowburn I Till K. What is it like to be a sport school student-athlete? A mixed method evaluation of holistic impacts and experiences. PLoS One. (2023) 18(11):e0289265. 10.1371/journal.pone.0289265 38033107 PMC10688867

